# High Prevalence of Syphilis among Young Pregnant Women in the Brazilian Amazon: A Cross-Sectional Study Based on Clinical Records in a Public Health Reference Unit in the City of Belém

**DOI:** 10.3390/pathogens13080686

**Published:** 2024-08-14

**Authors:** Ana Paula Figueiredo de Montalvão França, Camille Massena de Sousa, Misma Suely Gonçalves Araújo de Lima, Ricardo Roberto de Souza Fonseca, Rogério Valois Laurentino, Jacqueline Cortinhas Monteiro, Rosimar Neris Mantins Feitosa, Leonardo Miranda dos Santos, Aldemir Branco Oliveira-Filho, Luiz Fernando Almeida Machado

**Affiliations:** 1 Biology of Infectious and Parasitic Agents Post-Graduate Program, Federal University of Pará, Belém 66075-110, PA, Brazil; anapaula@alexandrohup.com (A.P.F.d.M.F.); chelseafc200966@hotmail.com (C.M.d.S.); mismaenf@yahoo.com.br (M.S.G.A.d.L.); 2 Virology Laboratory, Institute of Biological Sciences, Federal University of Pará, Belém 66075-110, PA, Brazil; ricardofonseca285@gmail.com (R.R.d.S.F.); valois@ufpa.br (R.V.L.); jacqueline@ufpa.br (J.C.M.); rosimar@ufpa.br (R.N.M.F.); 3 Bacteriology Laboratory, Institute of Biological Sciences, Federal University of Pará, Belém 66075-110, PA, Brazil; leonn_bio20@yahoo.com.br; 4 Study and Research Group on Vulnerable Populations, Institute for Coastal Studies, Federal University of Pará, Bragança 68600-000, PA, Brazil; olivfilho@ufpa.br

**Keywords:** *Treponema pallidum*, sexually transmitted infections, pregnant women, sexual and reproductive health

## Abstract

Background: Syphilis remains a significant global public health concern, and one of its consequences in pregnant women is the potential occurrence of congenital syphilis due to *Treponema pallidum* infection. This study determined the prevalence of syphilis among pregnant women undergoing prenatal care in a neighborhood on the outskirts of the city of Belém, Brazilian Amazon. Methods: This cross-sectional study used data from clinical records of 611 pregnant women who underwent prenatal care at a public health unit in 2019 and 2020. The reagent result for VDRL was used as an indicator of syphilis. Odds Ratio and chi-square tests were used to evaluate the association of information from pregnant women with syphilis. Results: The overall prevalence of syphilis was 5.2 % (32/611; 95 % CI: 3.5–7.0 %). Age under 23 years was identified as a risk factor for syphilis. Conclusions: The prevalence of syphilis among pregnant women in the outskirts of Belém is high, especially among younger women. There is an urgent need to intensify innovative sexual and reproductive health education initiatives and emphasize the importance of consistent practice of preventive measures against syphilis and other STIs in the Amazon region, especially in the young population.

## 1. Introduction

Syphilis is a Sexually Transmitted Infection (STI) caused by *Treponema pallidum* subspecies *pallidum*, a bacterium belonging to the *Spirochaetaceae* family, order *Spirochaetales*. This bacterium has a spiral shape, exclusively infects humans and has a highly invasive and immunoevasion profile. Its clinical manifestations are due to the inflammatory response caused by the replication of spirochetes in the tissues, and infected individuals normally follow a characteristic clinical evolution of this disease for a period equal to or greater than 10 years, when untreated [1,2].

Syphilis can be classified into phases, according to the symptoms presented, being called primary, secondary, latent and tertiary [2]. In addition to the phases already mentioned, congenital syphilis is usually devastating to the fetus if maternal infection is not detected and treated sufficiently early in the pregnancy. Most untreated primary and secondary syphilis infections in pregnancy result in severe adverse pregnancy outcomes, such as miscarriage or premature birth (10–40%), stillbirth (30–40%), hepatobiliary dysfunction (33–100%), cutaneous manifestations (40%), involvement of the Central Nervous System (40%), loss of vision, seizures and bone malformations (75%) [3].

The worldwide prevalence of congenital syphilis is estimated at 0.69%, resulting in a global rate of 4.73 cases per 1000 live births. It is more prevalent in poor countries, such as African countries, where its occurrence is 60% higher than the global estimate [4]. Although congenital syphilis is a notifiable disease and is included in prenatal screening programs, a form of screening in Brazil’s universal and decentralized health system, some gaps still make it impossible to eliminate [5].

The detection rate of syphilis in pregnancy increased between 2008 and 2018 in Brazil. The South region showed the highest trend, whereas the Midwest region presented the lowest trend [6]. In 2021, the occurrence of 7.7 cases of syphilis during pregnancy per 1000 live births was observed in Brazil [6], a result far from the target proposed by the WHO, which is 0.5 cases per 1000 live births [7]. In the period from 2005 to June 2023, 624,273 cases of syphilis were reported in pregnant women in Brazil, of which 10.3% were residents of the North region. In 2023, 4.123 cases of syphilis were reported in pregnant women in the North region, with the largest number of cases being observed in the state of Pará (1.577; 38.2%) [8].

According to the recommendations of the Brazilian Ministry of Health, every pregnant woman must be tested twice for syphilis during prenatal care (the first in the first trimester of pregnancy and the second in the third trimester), and sexual partners must also be tested. Furthermore, investigation for syphilis is mandatory after admission to the maternity ward for childbirth or in the event of an abortion. Therefore, the use of rapid tests for syphilis to investigate syphilis in prenatal care and in maternity wards is indicated by the advantages of speeding up diagnosis and treatment and optimizing the use of beds in maternity wards [9,10]. Research developed with a focus on prenatal care, diagnosis and treatment of syphilis during pregnancy helps to understand the socio-epidemiological aspects of this infection and can support the mobilization of future screening schemes and the reduction in the occurrence of congenital syphilis [11]. The present study aimed to estimate the prevalence and epidemiological indicators for syphilis among pregnant women who sought care in primary public health care centers in the city of Belém, Brazilian state of Pará, Amazon region.

## 2. Materials and Methods

### 2.1. Study Design, Ethics and Study Area

This cross-sectional study accessed and analyzed data from the clinical records of pregnant women who underwent prenatal care and routine consultations at the public health unit (PHU) in the Tapanã neighborhood in the city of Belém, state of Pará, Brazilian Amazon, from January 2019 to December 2020. This PHU provides basic and comprehensive care to the population of the Tapanã neighborhood and twelve other peripheral neighborhoods in the city of Belém. Basic health specialties, such as gynecology and obstetrics, are offered from Monday to Friday from 7 a.m. to 7 p.m. On the other hand, emergency care, such as urgency and emergency, occurs every day of the week, 24 h a day, in this PHU. The present study was approved by the Research Ethics Committee of the Institute of Biological Sciences at the Federal University of Pará (UFPA) under protocol number 3.297.951.

### 2.2. Sampling

This study used a non-probabilistic, intentional and conventional sample of pregnant women aged 18 or over attended at the reference public health unit in the city of Belém. The clinical records of these women were accessed, and all their information was organized and presented here. The variables considered were age, number of pregnancies, complaints about abortion, pre-eclampsia, beginning of sexual activity, prenatal care and information about childbirth and newborns. Records that did not present the results of the Venereal Disease Research Laboratory (VDRL) test were excluded. Reactive VDRL was considered positive for infection and non-reactive for negative infection, considering that treponemal tests were not always available at the Health Unit.

### 2.3. Statistical Analysis

The data were analyzed using the Statistical Package for Social Sciences (SPSS) version 21.0 (SPSS, Chicago, IL, EUA). The Odds Ratio and Chi-square tests were used to examine the degree of association between the prevalence of infections by syphilis in pregnancy and the different variables. The 95% confidence interval (CI) was calculated for all comparisons, and a *p* ≤ 0.05 significance level was considered for all the analyses.

## 3. Results

From January 2019 to December 2020, 750 pregnant women were treated at PHU Tapanã. All clinical records of these pregnant women were accessed and evaluated. However, 139 of them were excluded for presenting insufficient information. Therefore, six hundred and eleven pregnant women and their clinical information constituted the sample of this study. The overall prevalence of syphilis was 5.2% (32/611; 95% CI: 3.5–7.0%). The median age was 25 years (interquartile range: 21.0–30.0 years; range: 15–49 years). On the other hand, the median age of women who tested positive for syphilis was 23 years (interquartile range: 21.75–27.25 years; range: 18–34 years). The risk of syphilis is higher in pregnant women aged 23 years or younger (OR = 2.22; 95% CI = 1.07–4.64). The epidemiological characteristics of the pregnant women investigated in the study are presented in Table 1.

## 4. Discussion

In this study, a 5.2% prevalence of syphilis was found in pregnant women who sought prenatal care from public health services in a low-income neighborhood in the city of Belém, Pará, Amazon region of Brazil. The prevalence found in this study was lower than that found in pregnant women in other regions of Brazil, such as Juiz de Fora, Minas Gerais (9.61%) [12] and Porto Alegre, Rio Grande do Sul (11.0%) [13]; however, it was higher than that described in pregnant women in São Paulo (2.74%) [14] and in women assisted by primary health care units in Dourados, Mato Grosso do Sul (6.04%) [15] and was also higher than that found in pregnant women from other countries, such as Ethiopia (1.1–2.9%) [16,17], Togo (0.6%) [18], Malawi (1.92%) [19] and Tanzania (1.2%) [20].

Public health care in Brazil occurs through its Unified Health System (SUS), whose services are established through a decentralized and hierarchical network at different levels of complexity. Pregnant women receive prenatal care through basic care that operates in accordance with the Family Health Strategy Program (FHSP) in PHUs, which have doctors, nurses, nursing assistants and community health agents [21]. Most Brazilian pregnant women are low-income and only become aware of the diagnosis of syphilis after using these services, which is why it is worth considering the underreporting of syphilis during pregnancy in cases of low adherence to this program [22].

Primary health care in Brazil diagnoses and treats syphilis during pregnancy with a population coverage of up to 76.1% through rapid tests (treponemal test), easy to operate and perform, performed at the point of care and confirmed by non-invasive treponemal tests such as VDRL [5]. Although all international guidelines and recommendations for the prenatal monitoring of syphilis are available in Brazil, the rates of syphilis during pregnancy and congenital have increased considerably in the last decade [23]. The causes of the high rates of this infection can be explained by the lack of monitoring and adjustments of public policies on health education, prevention and treatment of syphilis with the increased capacity to detect this infection in the sexually active young population [24,25].

It was observed in this study that pregnant women aged 23 years or less are more likely to acquire syphilis compared to older than 23 years, and this behavior has been widely reported in young women in the reproductive period in all regions of Brazil [26,27]. Risky sexual behavior and poverty are factors that often accompany high rates of syphilis in pregnant women [28]. As syphilis cases are generally concentrated on the outskirts of large cities [22], to carry out this study, a UBS located in a neighborhood of a large Amazonian metropolis was selected, which is marked by being quite populated and presenting great socioeconomic inequalities [26]. In this way, this neighborhood would serve as a sentinel analysis of the management profile of public actions to combat syphilis during pregnancy and congenital syphilis in poor neighborhoods in the city of Belém and for understanding epidemiological indicators.

Congenital syphilis is the most serious outcome of syphilis during pregnancy. Despite being a preventable condition, the high rate of syphilis during pregnancy recorded here indicates flaws in the public system for tracking and treating syphilis in pregnant women. Congenital syphilis has been a mandatory notification for almost four decades in Brazil; however, its increase is recorded in all regions of this country [8]. After the first proposal to eliminate congenital syphilis in 1995 by resolution CE 116.R3 of the Pan-American Health Organization (PAHO), other attempts towards this same objective were unsuccessful in Brazil, even after the well-designed protocol to eliminate vertical transmission of congenital syphilis. In 2006 and 2021, the target of up to 1 case reported per 1000 live births per year was not achieved [6].

In Brazil, the health system still has gaps that keep it from achieving the level of elimination of syphilis during pregnancy as in the countries mentioned above [8], such as the large territorial extension, low demographics and high social vulnerability mainly in the North region, in addition to the low availability of basic prenatal care services, which is a consequence of the lack of health and STI prevention services and policies. Failures in health management in political-ideological contexts are among the main systematic causes that make the organization, expansion and territorialization of basic health services unfeasible [21]. Here, the lack of data in clinical records was a relevant gap for the consistent identification of the epidemiological scenario of syphilis during pregnancy. This gap must be urgently resolved by the public health service so that the service itself can record the characteristics of the people who are treated, as well as identify health problems and seek solutions to reduce their impacts.

The diagnosis and/or treatment of syphilis during pregnancy has weaknesses that prevent the expected benefits, such as avoiding or reducing congenital syphilis and its consequences. This study found that syphilis is high among women treated in one of the largest public health units in the city of Belém, in the Amazon region. Although worrying, these data corroborate records in the north of Brazil that present inadequate conditions in the provision of these services and that, over three decades, essential public health services in the Amazon were unable to reach the entire population with the expected quality [28]. Furthermore, the lack of basic knowledge about STI prevention in young populations in Amazonas, the low demand for services and the vulnerabilities of the health system revealed in the COVID-19 pandemic prove to be one of the possible influencers of the growing number of cases of STIs in our population because they led to a reduction in prenatal care [29]. One suggestion to help reduce the number of cases of syphilis and other STIs is the presentation and safe discussion of topics related to sexual and reproductive health in the school environment to deconstruct taboos, eliminate doubts and promote the health of adolescents and young people, as carried out in public schools in the city of Belo Horizonte [30].

This study has several limitations that should be considered. The use of secondary data and the occurrence of incomplete or lost clinical records made it impossible to carry out multivariate analyses and delve deeper into sexual and reproductive health (SRH) issues, such as age at first sexual intercourse, sexual education, identification and treatment of syphilis and other STIs. This information would facilitate the discussion of important aspects related to SRH and syphilis during pregnancy. To mitigate bias, analyses were performed separately for each variable. The absence of confirmatory and titration tests for syphilis is another limiting factor, which may overestimate the seroprevalence of syphilis and make it impossible to describe active syphilis. Furthermore, the sample of pregnant women participating in the study is restricted to a large peripheral region of the city of Belém but may not yet represent the city’s general population. Despite this, the information contained in this study can serve as a parameter for evaluating public policies aimed at combating syphilis during pregnancy in the Brazilian state of Pará, Amazon region, and other underdeveloped locations.

## 5. Conclusions

The prevalence of syphilis during pregnancy in a peripheral area of the city of Belém is high, and younger women are the most affected. This demonstrates non-compliance with the guidelines established by the Brazilian Ministry of Health regarding the diagnosis of syphilis. Therefore, we recommend additional studies to verify the situation of syphilis during pregnancy in other populations in the Amazon region, as well as the intensification of prenatal screening services and the quality of clinical control aimed at pregnant women with syphilis. Innovative actions in sexual and reproductive health and the importance of safety generated by the consistent use of preventive measures must be intensified among the young population of the city of Belém, the Amazon region and other underdeveloped locations.

## Figures and Tables

**Table 1:** Epidemiological characteristics of pregnant women (*n* = 611) treated at a public health unit on the outskirts of the city of Belém, Brazilian state of Pará, between 2019 and 2020.

Variables	Total *n* = 611 (%)	Syphilis + *n* = 32 (%)	*Odds Ratio*	CI 95% ^‡^	*p-Value* (OR) ^†^
Age (years)					
≤23	268 (43.8)	20 (7.4)	2.22	1.07–4.64	0.05
>23	343 (56.2)	12 (3.5)			
Sexual activity beginning (years)					
≤15	29 (4.7)	7 (24.1)	7.0	0.79–61.74	0.11
>15	47 (3.7)	1 (4.3)			
Not answered	535 (87.6)	24 (4.4)			
Abortions					
Yes	118 (19.3)	3 (2.5)	0.42	0.13–1.39	0.21
No	493 (80.7)	29 (5.8)			
Pregnancies					
1	227 (37.2)	14 (6.1)	1.34	0.65–2.74	0.54
>1	384 (62.8)	18 (4.6)			
Pre-eclampsia					
Yes	28 (4.6)	4 (14.2)	3.30	1.07–10.17	0.07
No	583 (95.4)	28 (4.8)			

VDRL with reagent result = Syphilis positive; ^‡^ Confidence Interval 95%; ^†^ Odds Ratio.

## Data Availability

The original contributions presented in the study are included in the article, further inquiries can be directed to the corresponding author.
